# Involvement in the tumor-infiltrating CD8^+^ T cell expression by the initial disease of remnant gastric cancer

**DOI:** 10.1186/s12957-022-02853-2

**Published:** 2022-11-30

**Authors:** Yoshihiko Kakiuchi, Satoru Kikuchi, Shinji Kuroda, Shunsuke Kagawa, Toshiyoshi Fujiwara

**Affiliations:** Department of Gastroenterological Surgery, Okayama University Graduate School of Medicine, Dentistry and Pharmaceutical Sciences, 2-5-1, Shikata-cho, Kita-ku, Okayama, 700-8558 Japan

**Keywords:** Remnant gastric cancer, Prognostic factor, Tumor-infiltrating lymphocytes, CD8^+^ T cell, Tumor immunity

## Abstract

**Background:**

Remnant gastric cancer (RGC) has been increasing for various reasons such as a longer life span, medical progress, and others. It generally has a poor prognosis, and its mechanism of occurrence is unknown. The purpose of this study was to evaluate the clinicopathological features of and clarify the oncological features of RGC.

**Methods:**

Between January 2002 and January 2017, 39 patients with RGC following distal gastrectomy underwent curative surgical resection at the Okayama University Hospital; their medical records and immunohistochemically stained extracted specimens were used for retrospective analysis.

**Results:**

On univariate analysis, initial gastric disease, pathological lymph node metastasis, and pathological stage were the significant factors associated with poor overall survival (*p*=0.014, 0.0061, and 0.016, respectively). Multivariate analysis of these 3 factors showed that only initial gastric disease caused by malignant disease was an independent factor associated with a poor prognosis (*p*=0.014, hazard ratio: 4.2, 95% confidence interval: 1.3–13.0). In addition, tumor-infiltrating CD8^+^ T cells expression was higher in the benign disease group than in the malignant group (*p*=0.046).

**Conclusions:**

Initial gastrectomy caused by malignant disease was an independent poor prognostic factor of RGC, and as one of the causes, lower level of tumor-infiltrating CD8^+^ T cells in RGC may involve in.

**Supplementary Information:**

The online version contains supplementary material available at 10.1186/s12957-022-02853-2.

## Background

Remnant gastric cancer (RGC) has been increasing with the evolution of medical technology, medical diagnostics, and longer life expectancy. RGC is defined as gastric cancer in the remnant stomach after partial gastrectomy for benign or malignant disease [1, 2]. The rate of RGC has generally been reported as 1–3% [3, 4], one of the less frequent cancers. However, especially in Eastern Asia, there is a high incidence of gastric cancer, and there is a possibility of a further increase in the future for the above reasons [2]. RGC is considered to have a different form of development from primary gastric cancer [5], and many details are unclear [6–8]. Moreover, it is difficult to perform a randomized, controlled trial because it is unknown when and who will develop the cancer after surgery. Therefore, there has been no improvement in prognosis over the past two decades, and the prognosis of RGC remains poor in comparison with that of primary gastric cancer [9, 10]. Thus, the aim of the present study was to evaluate the characteristics of RGC based on its clinicopathological features and clarify the oncological features for RGC based on the cases treated at our institution. Non-curative resection is excluded because it is well-known in the cancer treatment field that it does not improve the patients’ prognosis.

## Methods

### Patients

In this study, RGC was defined in accordance with the Japanese Classification of Gastric Carcinoma (English edition, ver. 3) [2]. Between January 2002 and January 2017, 39 patients with RGC following distal gastrectomy underwent curative surgical treatment at the Department of Gastroenterological Surgery, Okayama University Hospital. This study included only curative treatment, not non-curative treatment. Medical records of all patients were obtained from the hospital database. Preoperative factors (age, sex, prognostic nutritional index [PNI, X=10 × serum albumin + 0.005 × total peripheral lymphocyte count [11] calculated by blood test results, comorbidity, cause of initial gastrectomy and reconstruction methods in initial gastrectomy), tumor factors (histopathological data and status of remnant stomach), and postoperative factors (follow-up period, adjuvant therapy and recurrence) were examined retrospectively. Charlson Comorbidity Index was used for the assessment of patient’s comorbidity and the score was categorized as ≥2 and ≥1 referred to previous report [12]. The cause of initial gastrectomy and reconstruction methods in initial surgery were categorized as benign and malignant disease, and Billroth-I and others (Billroth-II and Roux-en Y), respectively. Tumor locations were categorized as anastomotic site and non-anastomotic site. Depth of invasion was categorized as T1 (mucosa or submucosa) or T2/3/4 (muscularis propria, subserosa, serosa-exposed, or serosa-infiltrating). Lymph node metastasis was categorized as negative or positive. Pathological stage was categorized as stage I or stage II/III (there were no stage IV cases). Histological types were categorized as differentiated type (well-differentiated, moderately differentiated, or papillary) or undifferentiated type (poorly differentiated, signet-ring cell carcinoma, or mucinous). Lymphatic invasion and venous invasion were categorized as negative or positive. All histopathological information including Sydney system was evaluated with the removed stomach with RGC by pathologist and determined in accordance with the Japanese Gastric Cancer Treatment Guidelines 2021 (6^th^ Edition) [13].

### Postoperative follow-up

Patients were followed-up every 3–6 months with physical examinations and laboratory blood tests. Patients underwent computed tomography (CT) every 6 months and esophagogastroduodenoscopy every 1 year.

### Immunohistochemical staining and assessment

Formalin-fixed, paraffin-embedded tissue samples cut at a thickness of 2 μm were deparaffinized and soaked in 0.3% H_2_O_2_ for 10 min at room temperature to extinguish endogenous peroxidase activity. After antigen retrieval by heating in a sodium citrate buffer solution or EDTA using a microwave, the samples were incubated with primary antibodies against CD8 (eBioscience, San Diego, CA, USA) and CD4 (eBioscience) overnight at 4 °C, and then with peroxidase-linked secondary antibody for 30 min at room temperature. After washing, the samples were stained with 3,3′-diaminobenzidine (Dako, Glostrup, Denmark) for visualization, and counterstained with Meyer’s hematoxylin. Immunohistochemical staining of CD8, CD4, FoxP3, and CD20 was performed in the stomach with RGC, and the stained area in the sample was measured using ImageJ software (National Institutes of Health, Bethesda, MD, USA). The assessment followed staining was that three different randomly selected fields were picked up and the average index of expression was calculated by Image J. Finally, the patients were classified into two groups with the median value as the cutoff value. The definition for tumor-infiltrating lymphocytes (s) was in accordance to our previous study [14, 15].

### Statistical analysis

All statistical analyses were performed using JMP software version 14.2 (SAS Institute, Cary, NC, USA). Fisher’s exact test was used for categorical variables, and the Mann-Whitney *U* test was used for continuous variables. The Kaplan-Meier method was used to estimate overall survival (OS) in each group, and survival rates were compared using the log-rank test. A probability (*P*) value less than 0.05 was considered significant.

## Results

### Clinicopathological features of RGC (Table 1)

The clinicopathological characteristics of the 39 patients are summarized in Table 1. Median age and PNI were 75 (interquartile range [IQR]: 71–79) years and 47.3 (IQR: 44.6–51.6), respectively. The median value of the Charlson Comorbidity Index was 0 (IQR: 0–2). Thirty-four (87.2%) patients were males, and 5 (12.8%) were females. Twenty benign diseases (51.3%) and 19 malignant diseases (48.7%) caused the initial gastrectomy. Reconstruction methods in initial gastrectomy were 20 cases (51.3%) of Billroth-I reconstruction and 19 cases (48.7%) of others. The mean interval from the initial gastric surgery to the RGC surgery was 28 years (IQR: 14.0–47.0 years). Neoadjuvant chemotherapy was performed in only 1 case (2.6%).

**Table 1:** Clinicopathologic features in the 39 patients with RGC

Age, years old	
Median (IQR)	75 (71–79)
Gender	
Male	34 (87.2%)
Female	5 (12.8%)
PNI	
Median (IQR)	47.3 (44.6–51.6)
Charlson Comorbidity Index	
Median (IQR)	0 (0–2)
Cause of initial gastrectomy	
Benign	20 (51.3%)
Malignant	19 (48.7%)
Reconstruction in initial gastrectomy	
Billroth-I	20 (51.3%)
Others	19 (48.7%)
Interval, years	
Median (IQR)	28 (14.0–47.0)
Neoadjuvant chemotherapy	
−	38 (97.4%)
+	1 (2.6%)
pT	
1	15 (38.4%)
2	4 (10.3%)
3	11 (28.2%)
4	9 (23.1%)
pN	
0	30 (76.9%)
1	6 (15.4%)
2	3 (7.7%)
pStage	
I	19 (48.7%)
II	13 (33.3%)
III	7 (18.0%)
Histological type	
Differentiated type	25 (64.1%)
Undifferentiated type	13 (33.3%)
Unknown	1 (2.6%)
Lymphatic invasion	
−	12 (30.8%)
+	27 (69.2%)
Venous invasion	
−	13 (33.3%)
+	26 (66.7%)
Tumor location	
Anastomosis	17 (43.6%)
Others	22 (56.4%)
Clavien-Dindo grade	
0 or I	20 (51.3%)
II	13 (33.3%)
III	3 (7.7%)
IV	3 (7.7%)
Adjacent organ resections	
−	34
+	5
Follow-up period, years	
Median (IQR)	4 (2.1–5.8)
Adjuvant chemotherapy	
−	32 (82.1%)
+	7 (17.9%)
Recurrence	
−	30 (76.9%)
+	9 (23.1%)

On histopathological examination, a large number were advanced cancers (61.6%), meaning that they were diagnosed as T2/3/4, but there were also many cases without lymph node metastasis (76.9%); as a result, pStage I (48.8%) accounted for approximately half of the cases. Differentiated type was the most common type (64.1%), and lymphatic and venous invasion-negative cases accounted for 30.8% and 33.3%, respectively. The tumor location was at the anastomotic site in 17 cases (43.6%). Patients who underwent Billroth-I reconstruction methods in initial gastrectomy showed RGC significantly more frequent in an anastomotic site than a non-anastomotic site (*p*=0.026) (Additional file 1).

Clavien-Dindo grade 0 or I, II, III, and IV of post-operative RGC were 20 (51.3%), 13 (33.3%), 3 (7.7%), and 3 (7.7%). Adjacent organ resections were performed in 5 patients (12.8%). Adjuvant chemotherapy was performed in 7 patients (17.9%), and 4 patients had completed the treatment. The median follow-up period of the 39 patients was 4 (IQR: 2.1-5.8) years. To identify differences in the patient background over time, they classified three groups as 2002–2007 (1^st^ period), 2008–2012 (2^nd^ period), and 2013–2017 (3^rd^ period). Only the initial disease showed significant differences in the three groups (*p*=0.0042); other backgrounds which included a 5-year OS rate did not (Additional file 2).

### Poor prognostic factor for RGC (Table 2)

**Fig. 1:**
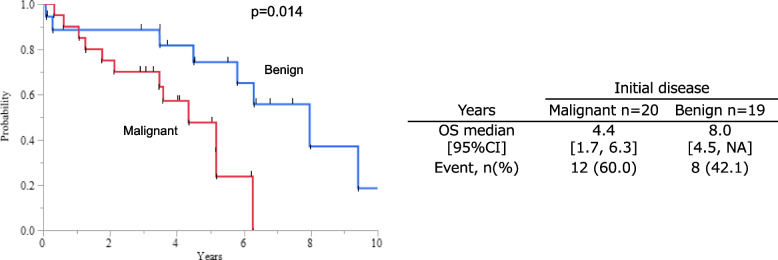
Kaplan-Meier plots of OS according to initial disease

The 5-year OS and relapse-free survival (RFS) rate was 60.7% and 74.7%, respectively (Additional file 3). The univariate analysis showed that malignant initial gastric disease, pathological lymph node metastasis positive, and pathological Stage ≥ II were significantly associated with a poor prognosis. Furthermore, the multivariate analysis including these 3 factors, which were *p*<0.05 in univariate analysis, showed that malignant initial gastric disease (hazard ratio [HR]: 4.2, 95% confidence interval [CI]: 1.3–13.0, *p*=0.014) was independently associated with a poor prognosis; there was a difference of over 40 months in median OS between those with and without the malignant initial disease (Fig 1).

**Table 2:** Predictors of overall survival: univariate and multivariate analyses

		Univariate analysis	Multivariate analysis		
		***p*** value	***p*** value	HR	95%CI
Background	Gender, male (vs female)	0.19			
	Tumor location, anastomosis (vs others)	0.96			
	Initial gastric disease, malignant (vs benign)	0.014	0.014	4.2	1.3–13.0
	Reconstruction at initial gastrectomy, Billroth-I (vs others)	0.34			
	Charlson Comorbidity Index, ≥2 (vs ≥1)	0.31			
Tumor	pT, ≥2 (vs 1)	0.063			
	pN, + (vs −)	0.0061	0.2		
	pStage, ≥II (vs I)	0.016	0.095		
	Histology, undifferentiated type (vs differentiated type)	0.17			
	Ly, + (vs −)	0.52			
	V, + (vs −)	0.31			
Pre- and postsurgery	Clavien-Dindo grade, ≥III (vs II≥)	0.37			
	Adjacent organ resections, + (vs −)	0.57			
	Adjuvant chemotherapy, + (vs −)	0.9

### Clinicopathological differences between benign and malignant initial gastric disease (Table 3)

Next, we assessed the clinicopathological differences between benign and malignant initial gastric disease, and Table 3 shows the relationship of each factor. In the patient background, the male was significantly more in the benign group than the malignant group (*p*=0.047). The interval between initial and RGC surgery was significantly longer for the benign group than the malignant group (*p*<0.001), though there was no difference in the age at RGC surgery. Other background characteristics, tumor, and surgical factors, which was also including advanced tumor factors and postoperative complications, were not associated with the initial disease. Furthermore, lymph node metastasis of each station did not show significant differences between benign and malignant initial disease whereas the benign initial disease group tended to have more extensive metastasis-positive lymph node stations than the malignant (Additional file 4).

**Table 3:** Relationship of initial disease with each factor

		Benign	Malignant	***p*** value
Background	Age, median (IQR)	73.6	74.7	0.45
	Sex (male/female)	19/0	15/5	0.047
	Tumor location (anastomosis/others)	12/7	7/13	0.11
	Interval, median (IQR)	47 (32–50)	14.5 (5.75–23.25)	<0.001
	Reconstruction at initial gastrectomy (Billroth-I/others)	8/11	12/8	0.34
Tumor	pT (1/≥2)	9/10	6/14	0.33
	pN (−/+)	14/5	16/4	0.72
	pStage (I/≥II)	10/9	9/11	0.75
	Histology (differentiated/undifferentiated type)	14/4	11/9	0.18
	Ly (−/+)	6/13	6/14	1
	V (−/+)	8/11	5/15	0.32
Post-surgery	Clavien-Dindo grade (≤II/≥III)	15/2	16/4	0.67
	Adjacent organ resections,(−/+))	18/1	16/4	0.34
	Adjuvant chemotherapy (−/+)	17/2	15/5	0.41
Sydney system	Inflammation (+/−)	18/1	19/1	1
	Activity (+/−)	9/10	4/16	0.096
	Atrophy (+/−)	16/3	17/3	1
	Intestinal metaplasia (+/−)	7/12	5/15	0.50
	H.Pylori (+/−)	9/10	3/17	0.041
Immunology, area index	CD8	3562	2243	0.046
	CD4	30,419	16,614	0.15
	FoxP3	16,725	7689	0.20
	CD20	187,881	19,740	0.87

Furthermore, we analyzed the pathological assessment of the remnant stomach condition according to the histological division of the Sydney system, and of the immunological condition in RGC. In the stomach condition according to the Sydney system, *Helicobacter pylori* (*H. pylori*) infection was significantly more in the benign group than the malignant group (*p*=0.041), and in the other status, there was no difference between the two groups.

In the immunological condition in RGC, before the analysis, it was confirmed that the staining was completely successful and that there was a difference in expression levels for each tumor tissue. The tumor-infiltrating CD8^+^ T cell expression level in RGC was significantly higher following benign initial disease than following malignant initial disease (*p*=0.046), whereas the tumor-infiltrating expression of CD8, CD4, FoxP3, and CD20 was not directly relevance with RFS (Additional file 5).

## Discussion

RGC is often detected at an advanced stage, and the 5-year survival rate was reported to be poor in the previous studies [6, 16–18]. Part of the reason is that the resectability of RGC was low, the postoperative mortality was high, and surgeries were difficult [9, 19, 20]. In the initial gastrectomy, the omental bursa and greater omentum, in addition to the lymph node dissection, were removed in some cases. Therefore, strong adhesions were observed around the remnant stomach. If any morbidity, such as pancreatic fistula, occurred at the initial operation, it was more complicated. The other change caused by the initial surgery is lymphatic flow. Some adhesions around the remnant stomach form a new lymphatic course [16, 21]. The surgical difficulty such reasons caused the complications. In the present study, patients of the malignant initial disease had a poor prognosis than patients of the benign initial disease whereas postoperative complication, which was frequently considered a poor prognostic factor, was not a prognostic factor. On the other hand, remnant gastric status had some differences between the benign and malignant initial disease, which is impressive. The remnant stomach in the patient of benign initial disease was observed to be more infected with *H. pylori* than in the malignant initial disease. We considered the reason for this result that there was no concept of removing H.Pylori following the gastrectomy in the era when surgery for the benign disease was performed. Furthermore, as the postoperative follow-up, the patients of malignant initial disease had generally a better follow up, probably due to the oncological feature of the disease. Therefore H.Pylori eradication probably was more frequent in this group. This result suggested the importance of removing H.Pylori following gastrectomy.

Some reports showed T stage and venous invasion were important prognostic factors [22], whereas some studies identified differences in lymphatic flow between benign and malignant initial diseases [5, 23, 24]. In fact, the initial gastric disease was also a prognostic factor in the present study, which means that lymph node dissection at the initial surgery has some impact on the prognosis of RGC. With the change of lymph flow to the bloodstream, lymph node metastasis may develop more frequently than in the usual course and may easily metastasize beyond regional lymph nodes, to lymph nodes such as the paraaortic lymph nodes. It has already been reported that the frequency of lymph node metastasis in the splenic hilum and along the splenic artery is higher in RGC than in primary cancers of the upper third of the stomach [8, 9, 16, 25], and surely lymphatic dissection was justified [26]. However, although there were some reports of changes in the lymph node metastasis site with the lymph flow change, to our knowledge, there were no reports examining the change in lymph-related immunity, such as tumor-infiltrating lymphocytes (TILs). Interestingly, the present study showed that the tumor-infiltrating CD8+ T cells expression was higher in the benign initial disease than in the malignant initial disease. We could not clarify the reason yet, but we consider that it may be related to the change of lymph flow by lymph node dissection and the T cell exhaustion with the malignant initial disease [27]. In breast cancer patients, TILs expression is actually significantly associated with improved OS [28, 29]. Therefore, our results that the patients of malignant initial disease had low CD8^+^ T cells expression and poor prognosis of RGC may indicate that tumor immunity suppression is involved in the poor prognosis of RGC. This appears to be the first report showing that CD8^+^ T cells may be involved in RGC prognosis.

Although this study has provided some important information for clinical practice, it has several limitations. First, this was a single-center, retrospective study, and the first patient underwent RGC surgery 15 years ago, which is a very long time for a clinical study and may have decreased the study quality. However, the only change during this period has been that the mainly surgical approach shifted from open to laparoscopic surgery, and there has been no change including the criteria of lymph node dissection or other organ resections and surgical indications. Second, the sample size was small, involving only 39 patients.

## Conclusions

The prognosis of patients with RGC was independently associated with initial gastric disease, and tumor-infiltrating CD8^+^ T cells in RGC were expressed more in the benign initial gastric disease group than in the malignant initial gastric disease group. Generally, it is difficult to conduct a prospective, randomized trial in RGC; therefore, it is very meaningful to be able to predict the prognosis from preoperative or postoperative factors, and, among them, TILs analysis may be very important.

## Supplementary Information

**Additional file 1.** The association between reconstruction method in initial disease and tumor location in RGC.

**Additional file 2.** The differences of the background between 2002-2007, 2008-2012 and 2013-2017.

**Additional file 3.** Kaplan-Meier plots of OS and RFS. Dotted line indicates 5-year OS.

**Additional file 4.** The distribution of the lymph node metastasis between benign and malignant initial disease.

**Additional file 5.** (Left) Representative high/low expression images of tumor-infiltrating CD8, CD4, FoxP3 and CD20. (scale bar: 50 μm). (Right) The association between CD8, CD4, FoxP3 and CD20, and RFS. (Bottom) Distribution map of tumor-infiltrating CD8^+^ and CD4^+^ T cells. Dotted line indicates median values. The association between tumor-infiltrating expression levels of CD8, CD4, FoxP3 and CD20 and RFS.

## Data Availability

The datasets used during the current study are available from the corresponding author on reasonable request.
