# Supplementary figures and images for: Involvement in the tumor-infiltrating CD8+ T cell expression by the initial disease of remnant gastric cancer
Source: World J Surg Oncol. 2022 Nov 30;20:374. doi: 10.1186/s12957-022-02853-2 (PMC9713981; doi:10.1186/s12957-022-02853-2)

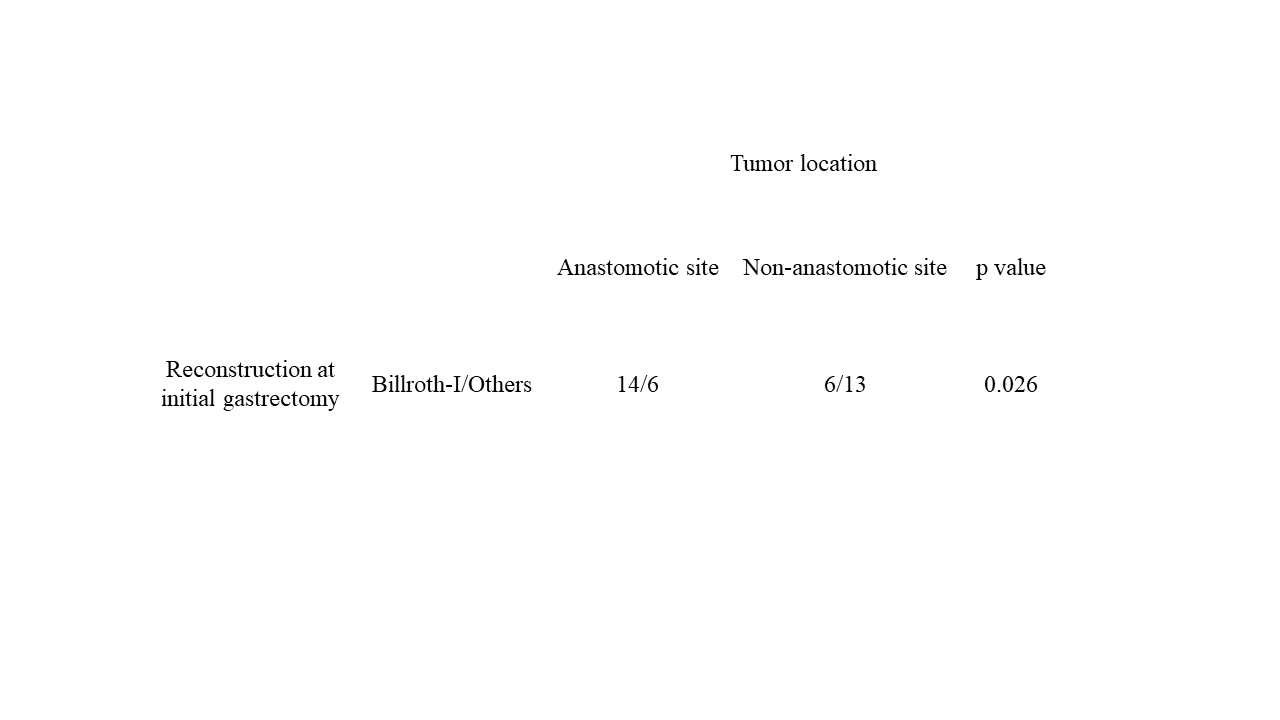

Supplement: Supplementary file 1 — Additional file 1. The association between reconstruction method in initial disease and tumor location in RGC. [file 12957_2022_2853_MOESM1_ESM.tif]

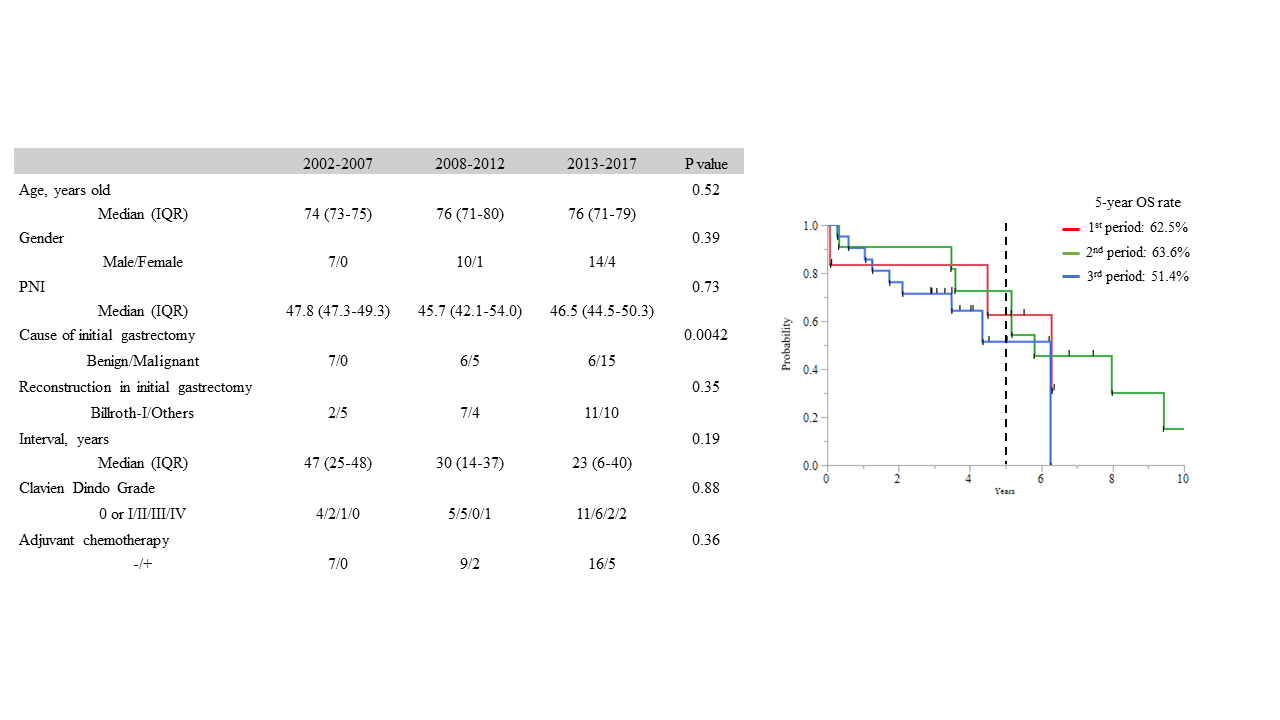

Supplement: Supplementary file 2 — Additional file 2. The differences of the background between 2002-2007, 2008-2012 and 2013-2017. [file 12957_2022_2853_MOESM2_ESM.tif]

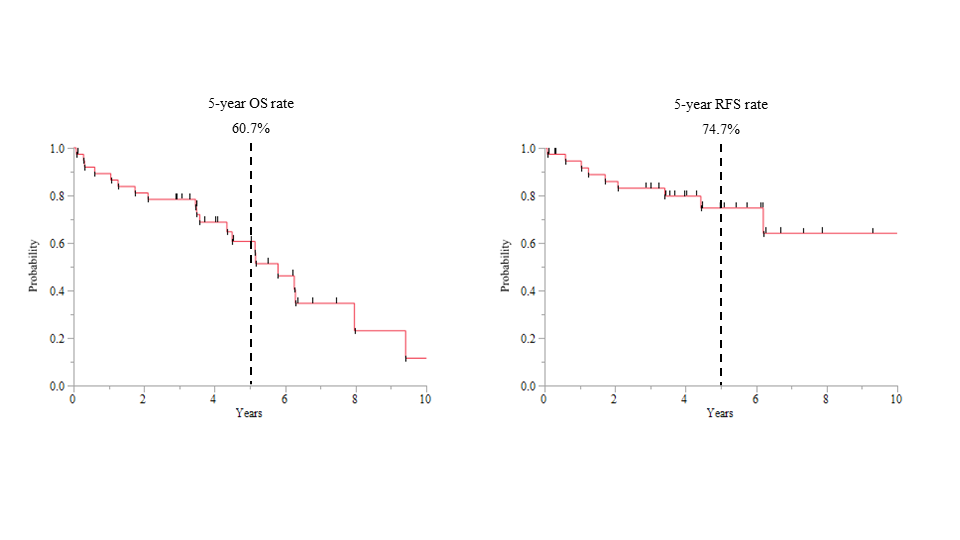

Supplement: Supplementary file 3 — Additional file 3. Kaplan-Meier plots of OS and RFS. Dotted line indicates 5-year OS. [file 12957_2022_2853_MOESM3_ESM.tif]

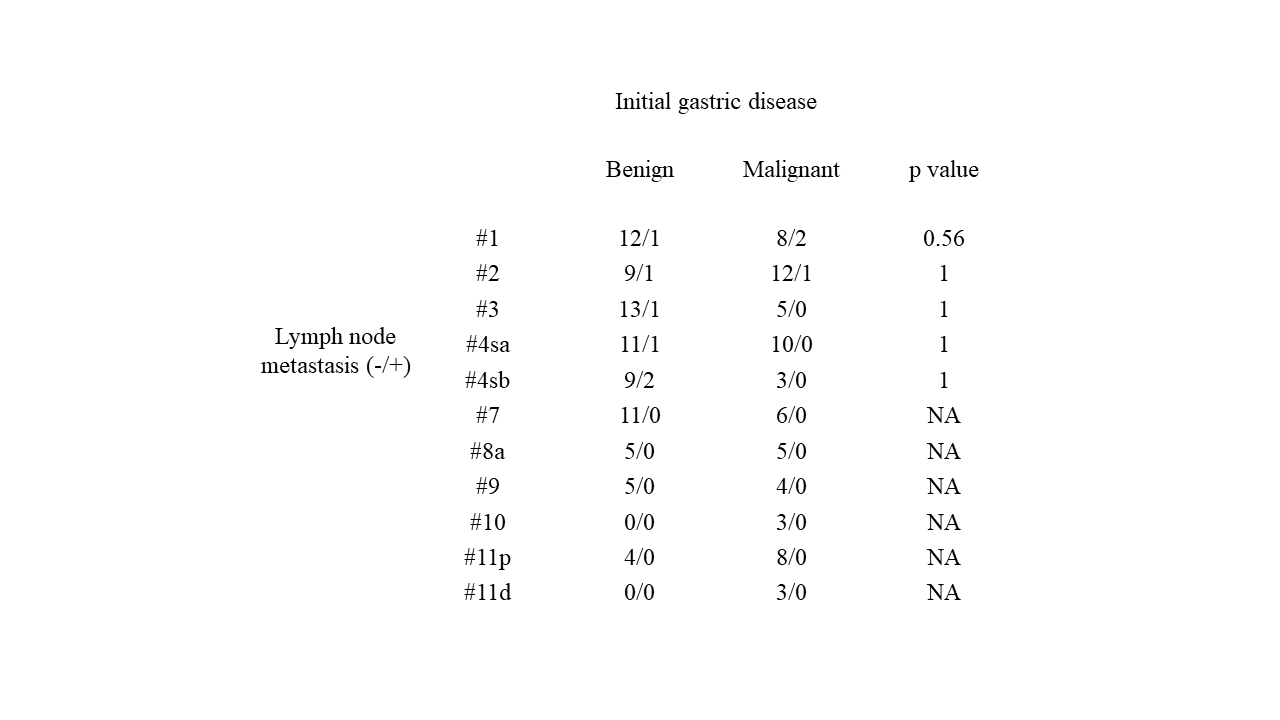

Supplement: Supplementary file 4 — Additional file 4. The distribution of the lymph node metastasis between benign and malignant initial disease. [file 12957_2022_2853_MOESM4_ESM.tif]

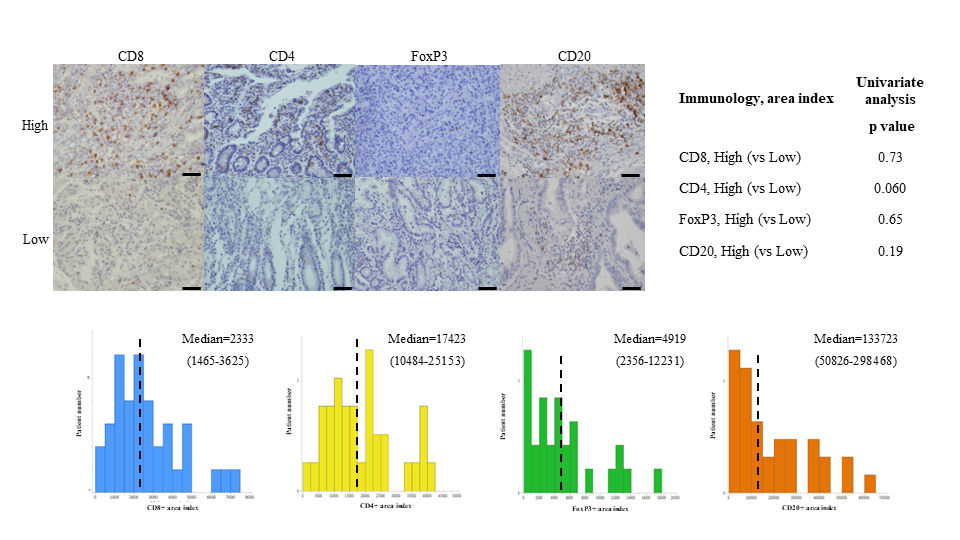

Supplement: Supplementary file 5 — Additional file 5. (Left) Representative high/low expression images of tumor-infiltrating CD8, CD4, FoxP3 and CD20. (scale bar: 50 μm). (Right) The association between CD8, CD4, FoxP3 and CD20, and RFS. (Bottom) Distribution map of tumor-infiltrating CD8+ and CD4+ T cells. Dotted line indicates median values. The association between tumor-infiltrating expression levels of CD8, CD4, FoxP3 and CD20 and RFS. [file 12957_2022_2853_MOESM5_ESM.tif]
